# Supplementary material for: Transcriptome and Physiological Analysis of Rapeseed Tolerance to Post-Flowering Temperature Increase
Source: Int J Mol Sci. 2023 Oct 26;24(21):15593. doi: 10.3390/ijms242115593 (PMC10648292; doi:10.3390/ijms242115593)
Supplement: Supplementary file 1 [file ijms-24-15593-s001.zip › Figure S1.pdf]

A

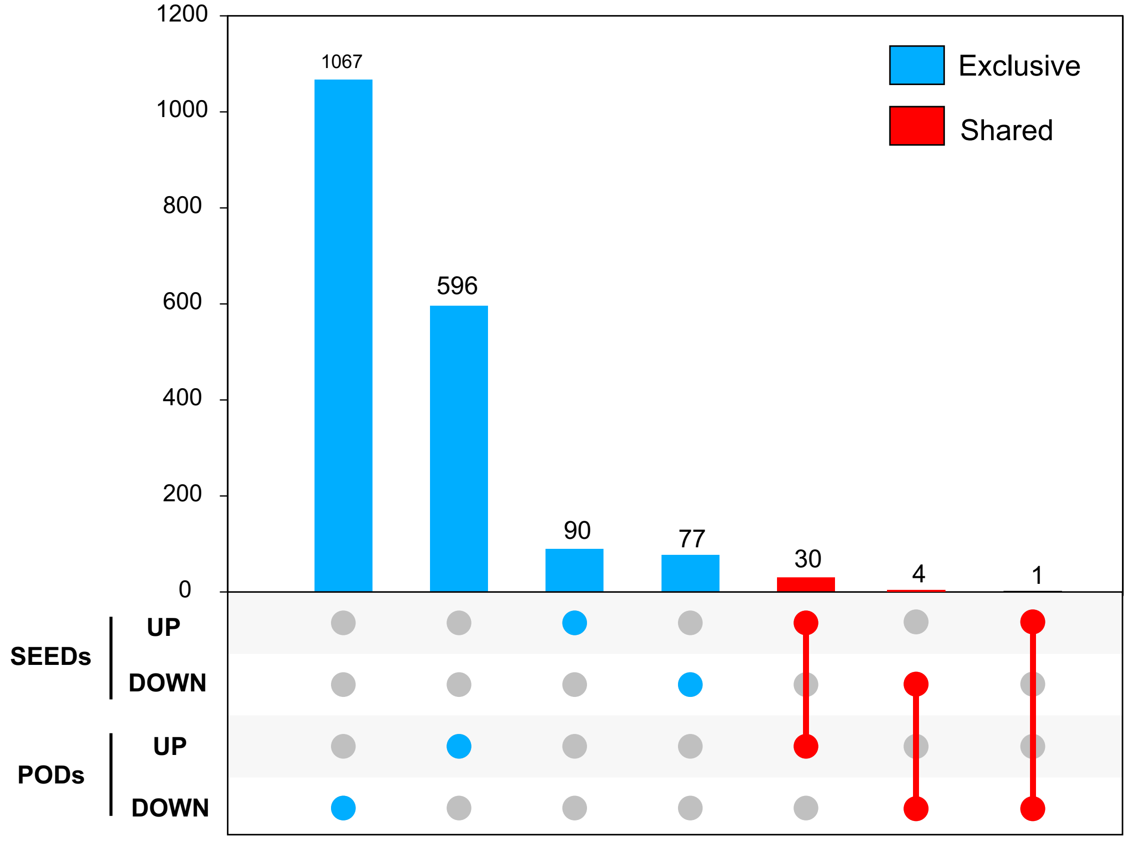

B

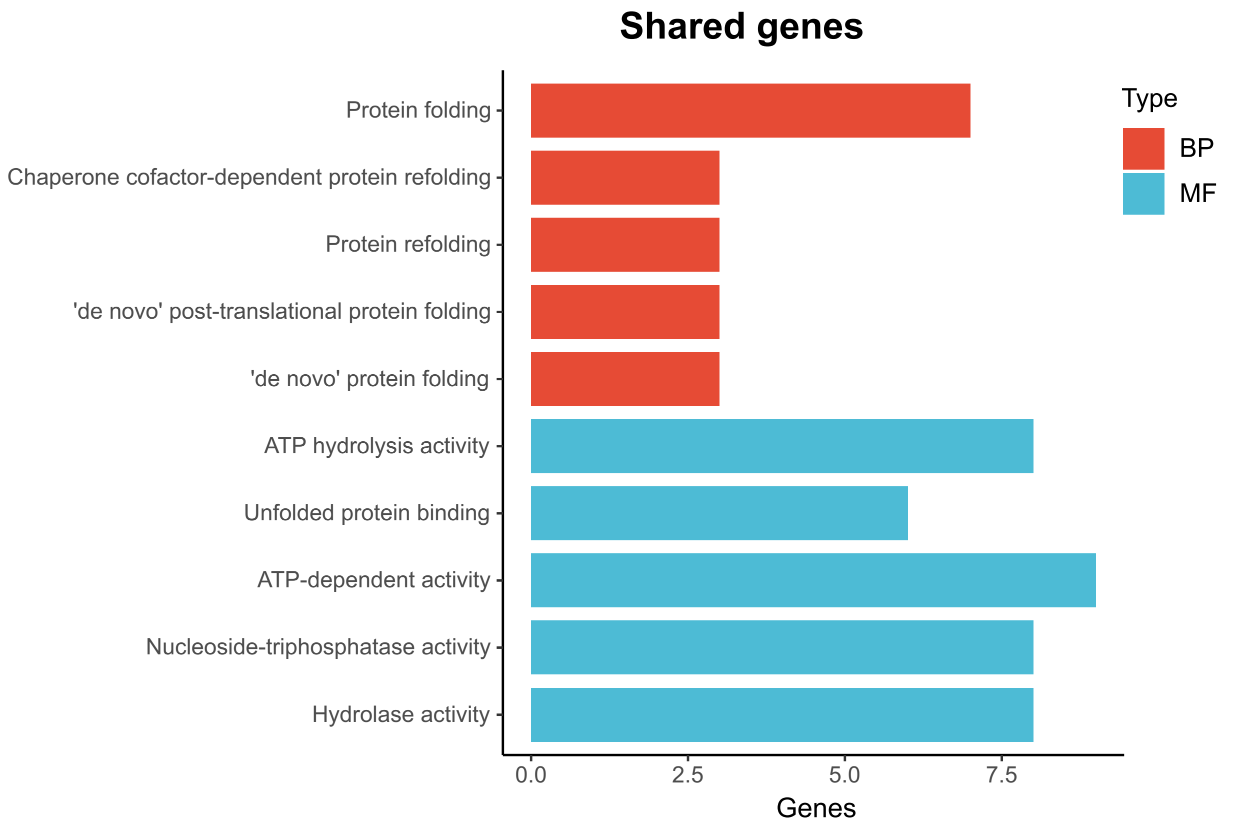

Figure S1: UpSet plots and Gene Ontology (GO) enrichment analysis of differentially expressed genes (DEGs) in seeds and siliques. (A) UpSet plot illustrating the intersections of DEGs identified in seeds and siliques. Each vertical bar represents a unique combination of DEGs, with the number of shared genes indicated by the size of the bar. (B) GO enrichment analysis of shared DEGs between seeds and siliques. The top significantly enriched biological process, molecular function, and cellular component GO terms are displayed.
