# Supplementary material for: Transcriptome and Physiological Analysis of Rapeseed Tolerance to Post-Flowering Temperature Increase
Source: Int J Mol Sci. 2023 Oct 26;24(21):15593. doi: 10.3390/ijms242115593 (PMC10648292; doi:10.3390/ijms242115593)
Supplement: Supplementary file 1 [file ijms-24-15593-s001.zip › Figure S2.pdf]

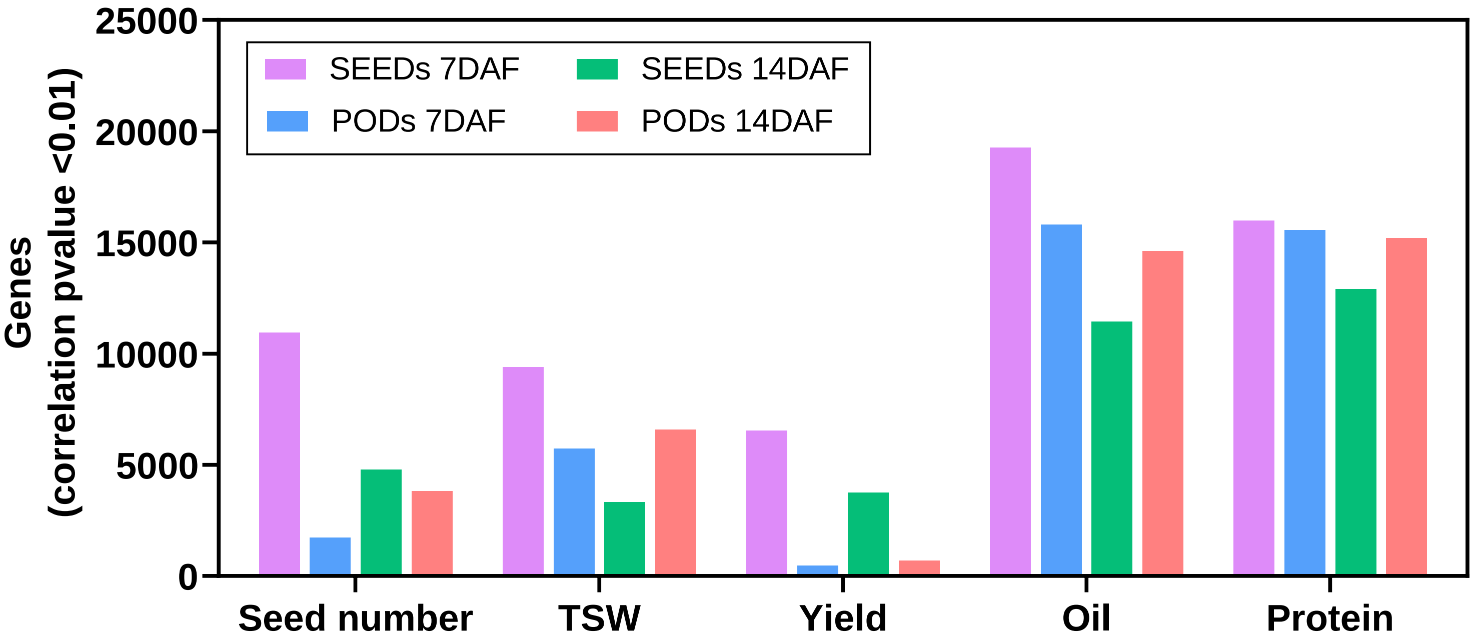

Figure S2: Bar plot illustrating the number of genes significantly correlated with agronomic traits of yield and quality in seed and silique networks at different developmental stages.
