# Supplementary material for: Transcriptome and Physiological Analysis of Rapeseed Tolerance to Post-Flowering Temperature Increase
Source: Int J Mol Sci. 2023 Oct 26;24(21):15593. doi: 10.3390/ijms242115593 (PMC10648292; doi:10.3390/ijms242115593)
Supplement: Supplementary file 1 [file ijms-24-15593-s001.zip › Figure S3.pdf]

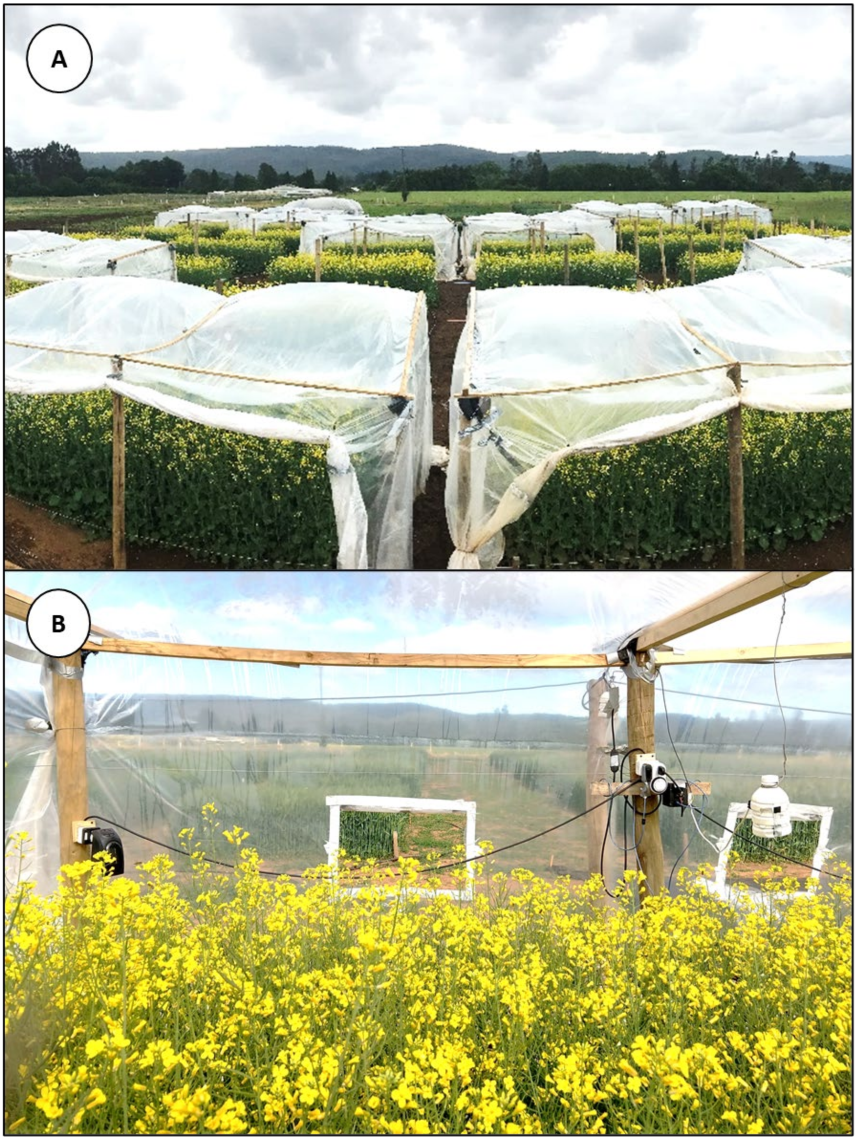

Figure S3. Experimental Design for Heat Stress Treatments During Seed Filling: (A) Depicts the establishment of plastic greenhouses over designated plots for controlled environmental manipulation; (B) Illustrates the interior of the greenhouse, highlighting the placement of thermo-fans and an automated temperature sensor to regulate and monitor heat increments.
