# Supplementary material for: Transcriptome and Physiological Analysis of Rapeseed Tolerance to Post-Flowering Temperature Increase
Source: Int J Mol Sci. 2023 Oct 26;24(21):15593. doi: 10.3390/ijms242115593 (PMC10648292; doi:10.3390/ijms242115593)
Supplement: Supplementary file 1 [file ijms-24-15593-s001.zip › Supplementary methods.pdf]

SUPPLEMENTARY METHODS

1. Experimental Set-Up and Crop Management

Two field experiments were conducted at the Austral Farming Experimental Station (EEAA) of the Universidad Austral de Chile, Valdivia, Chile, situated at 39° 47' S, 73° 14' W, utilizing a Duric Hapludand soil. Two spring rapeseed hybrids (Lumen and Solar from NPZ-Lembke, Germany) were evaluated under two distinct heat stress treatments: (i) an unaltered control, and (ii) a treatment with a 5°C temperature elevation initiated at the onset of flowering [BBCH 61] and sustained for 15 days post-flowering (DAF). The first experiment was sown on September 5, 2019 (season 1), while the second experiment was sown on September 4, 2020 (season 2). In the first experiment, a split-plot design with three replicates was employed, wherein the heat stress treatments were allocated to the main plots and the genotypes to the sub-plots. In contrast, the second experiment adopted a randomized block design with three replicates, where both genotypes and heat stress treatments were randomized within each block.

All experimental plots measured 2 m in length and 3.5 m in width and consisted of 11 rows. Rapeseed was sown at a seeding density of 55 plants m^{-2}. Optimal agronomic conditions were maintained throughout the experiments, with the exception of the imposed heat treatments. Nutrient management adhered to previously established protocols to obviate phosphorus, potassium, magnesium, and sulfur deficiencies [1]. Irrigation was surface-based and calibrated to local rainfall patterns to avert water stress. A comprehensive pest and disease management strategy was executed based on manufacturer-recommended guidelines to preclude biotic constraints [1].

2. Temperature Modulation in Treated Plots

Portable greenhouse chambers, constructed with wooden frames and covered with 100 μm-thick transparent polyethylene film [2], were employed to elevate temperatures in the heat-stress-treated plots (Figure S3). The apex of each structure was positioned 0.3-0.4 m above the plant canopy. To achieve the target 5°C temperature increase, automatic sensors were deployed both inside and outside these chambers, and thermo-fans with a 2000 W capacity were utilized. The radiation intercepted by the polyethylene film was quantitatively averaged at 10%.

3. Phenological and Physiological Assessments

Phenological progression was monitored bi-weekly using the BBCH scale specific to rapeseed [3]. Biomass samples were collected from one linear meter of the central rows in each plot at physiological maturity, which was determined when the seeds within the siliques attained a hard and darkened state (BBCH 89). Subsequent to oven-drying at 65°C for 48 h, siliques were threshed and weighed. Metrics including seed yield, seed number, thousand-seed weight, and quality traits such as seed oil and protein concentrations were either measured or calculated. Seed oil concentration was ascertained via Near Infrared Reflectometry (NIR) (Foss Infratec 1241, Hilleroed, Denmark), while seed nitrogen concentration was quantified using the Kjeldahl method [4]. Protein concentration in seeds was computed using a conversion factor of 5.8 [5].

Statistical analyses were performed employing a standard analysis of variance in Statgraphics Centurion 18. Differences among genotypes and between heat stress treatments were deemed statistically significant at a 5% probability level as determined by the least squares mean differences test. The normal distribution and homogeneity of residuals were verified for all collected data [6].

4. Meteorological Data

Meteorological parameters, specifically the maximum and minimum air temperatures along with the incidence of solar radiation (ISR), were continually recorded at 30-minute intervals from the time of sowing until harvest. Data were collected at the Austral Meteorological Station of the EEAA, located approximately 200 m from the experimental site (http://agromet.inia.cl/).

5. Supplementary References

1. Verdejo, J.; Calderini, D. F., Plasticity of seed weight in winter and spring rapeseed is higher in a narrow but different window after flowering. *Field Crop Res* **2020,** 250, 107777.
2. Lizana, X. C.; Calderini, D. F., Yield and grain quality of wheat in response to increased temperatures at key periods for grain number and grain weight determination: considerations for the climatic change scenarios of Chile. *J Agr Sci* **2013,** 151, (2), 209-221.
3. Meier, U., Growth stages of mono-and dicotyledonous plants. In *BBCH Monograph*, Julius Kühn-Institut: Quedlinburg, Germany, 2018; p 204 p.
4. Kirk, P. L., Kjeldahl Method for Total Nitrogen. *Analytical Chemistry* **1950,** 22, (2), 354-358.
5. Merrill, A. L.; Watt, B. K., *Energy value of foods: basis and derivation*. Slightly rev. ed.; Human Nutrition Research Branch, Agricultural Research Service; for sale by the Supt. of Docs., U.S. Govt. Print. Off.: Washington, 1973; p iv, 105 p.
6. Kutner, M.; Nachtsheim, C.; Neter, J., *Applied Linear Regression Models*. McGraw-Hill Education: Boston, 2004; p 701.
