# Supplementary material for: Transcriptome and Physiological Analysis of Rapeseed Tolerance to Post-Flowering Temperature Increase
Source: Int J Mol Sci. 2023 Oct 26;24(21):15593. doi: 10.3390/ijms242115593 (PMC10648292; doi:10.3390/ijms242115593)
Supplement: Supplementary file 1 [file ijms-24-15593-s001.zip › Table S3.pdf]

Table S3. Mean and maximum temperatures (T), incident solar radiation (ISR) and cumulative solar radiation during heat stress treatments across seasons.

Genotype	Thermal treatment	Tmean (°C)	Tmax (°C)	ISRmean $MJ\ m^{-2}\ day^{-1}$	Cumulative ISR $MJ\ m^{-2}$
**Season 1**					
Lumen	Control	14.1 ± 0.6	27.2	22.4 ± 1.7	358.7
	0-15 DAF	18.4 ± 0.6	31.5	20.2 ± 1.5	322.8
Solar	Control	14.7 ± 0.6	27.2	24.2 ± 1.5	386.6
	0-15 DAF	19.4 ± 0.6	31.9	21.7 ± 1.3	347.9
**Season 2**					
Lumen	Control	12.9 ± 0.5	23.7	21.3 ± 2.1	362.2
	0-15 DAF	17.5 ± 0.5	27.8	19.2 ± 1.9	326.0
Solar	Control	13.1 ± 0.6	27.5	21.6 ± 2.2	366.5
	0-15 DAF	17.7 ± 0.6	29.6	19.4 ± 2.0	329.9

Mean ± standard error.
