# Supplementary material for: Transcriptome and Physiological Analysis of Rapeseed Tolerance to Post-Flowering Temperature Increase
Source: Int J Mol Sci. 2023 Oct 26;24(21):15593. doi: 10.3390/ijms242115593 (PMC10648292; doi:10.3390/ijms242115593)
Supplement: Supplementary file 1 [file ijms-24-15593-s001.zip › Table S4.pdf]

Table S4. Dates of plant sampling for transcriptomic analysis for each genotype and developmental stage.

Year	Genotype	Date					
		Sowing	Emergence	BBCH61	Start heat stress	7 DAF	14 DAF
2019	Lumen	05-sep-19	13-sep-19	17-nov-19	18-nov-19	25-nov-19	02-dic-19
	Solar	05-sep-19	18-sep-19	22-nov-19	23-nov-19	30-nov-19	07-dic-19
2020	Lumen	02-Sep-20	18-Sep-20	18-Nov-20	19-Nov-20	26-Nov-20	03-Dec-20
	Solar	02-Sep-20	19-Sep-20	19-Nov-20	20-Nov-20	27-Nov-20	04-Dec-20

Year	Genotype	Days from last event					
		Sowing	Emergence	BBCH61	Start heat stress	7 DAF	14 DAF
2019	Lumen	0	8	65	1	7	7
	Solar	0	13	65	1	7	7
2020	Lumen	0	16	61	1	7	7
	Solar	0	17	61	1	7	7

End heat stress	PM	Harvest
03-dic-19	11-ene-20	31-ene-20
08-dic-19	10-ene-20	31-ene-20
04-Dec-20	13-Jan-21	25-Jan-21
05-Dec-20	06-Jan-21	25-Jan-21

End heat stress	PM	Harvest
1	39	20
1	33	21
1	40	12
1	32	19
